# DHFR Inhibitors: Reading the Past for Discovering Novel Anticancer Agents

**DOI:** 10.3390/molecules24061140

**Published:** 2019-03-22

**Authors:** Maria Valeria Raimondi, Ornella Randazzo, Mery La Franca, Giampaolo Barone, Elisa Vignoni, Daniela Rossi, Simona Collina

**Affiliations:** 1 Department of Biological, Chemical and Pharmaceutical Sciences and Technologies (STEBICEF), University of Palermo, via Archirafi 32, 90123 Palermo, Italy; ornella.randazzo01@unipa.it (O.R.); mery.lafranca@unipa.it (M.L.F.); giampaolo.barone@unipa.it (G.B.); 2 Drug Sciences Department, Medicinal Chemistry and Pharmaceutical Technology Section, University of Pavia, via Taramelli 12, 27100 Pavia, Italy; elisa.vignoni01@universitadipavia.it (E.V.); daniela.rossi@unipv.it (D.R.)

**Keywords:** dihydrofolate reductase (DHFR) enzyme, DHFR inhibitors as anticancer agents, heterocyclic compounds, DHFR drug discovery, hybrid compounds

## Abstract

Dihydrofolate reductase inhibitors are an important class of drugs, as evidenced by their use as antibacterial, antimalarial, antifungal, and anticancer agents. Progress in understanding the biochemical basis of mechanisms responsible for enzyme selectivity and antiproliferative effects has renewed the interest in antifolates for cancer chemotherapy and prompted the medicinal chemistry community to develop novel and selective human DHFR inhibitors, thus leading to a new generation of DHFR inhibitors. This work summarizes the mechanism of action, chemical, and anticancer profile of the DHFR inhibitors discovered in the last six years. New strategies in DHFR drug discovery are also provided, in order to thoroughly delineate the current landscape for medicinal chemists interested in furthering this study in the anticancer field.

## 1. Introduction

Since the middle of the last century, the potential of the dihydrofolate reductase (DHFR) enzyme as a therapeutic target for treating infections has been evidenced [1,2]. DHFR catalyzes the reduction of dihydrofolate to tetrahydrofolate using NADPH, and it is involved in the synthesis of raw material for cell proliferation, in both prokaryotic and eukaryotic cells. DHFR inhibitors are commonly used for fighting malaria and other protozoal infections, as well as for treating fungal, bacterial, and mycobacterial infections [3]. Over the years, several compounds have been discovered and different drugs have entered the market. Among them, we have to mention pyrimethamine and proguanil as antimalarial drugs [4,5]; trimethoprim, an antibacterial drug commonly used in association with sulfonamides, like sulfamethoxazole [6,7]; and methotrexate, the first-in-class anti-cancer agent acting via DHFR inhibition [8,9]. Methotrexate inhibits DHFR with a high affinity, thus reducing the amount of tetrahydrofolates required for the synthesis of pyrimidine and purines. Consequently, RNA and DNA synthesis is stopped and the cancer cells die. From a chemical standpoint, methotrexate shows several drawbacks, such as a poor solubility and relevant toxic side effects [10,11,12]. DHFR inhibitors are among the most used classes of anticancer agents and finding novel agents with a promising pharmacological profile still remains one of the major challenges for medicinal chemists, as testified by the literature trend of the last 20 years. 

In this review, after a brief overview of the physiological role of DHFR in cells and particularly in cancer cells, we focus on DHFR inhibitors for cancer therapy. Particularly, we highlight compounds already marketed and new scaffolds that could be relevant for anticancer therapy. 

## 2. Physiological Role and Structure of DHFR

**Figure 1:**
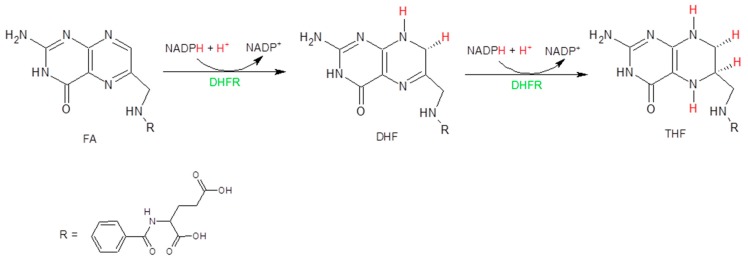
Reduction of FA in THF.

Folic acid (FA) is a water-soluble vitamin important for biological systems. It is not biologically active per se, but it is the precursor of the active form known as tetrahydrofolate (THF), which is essential for the de novo synthesis of purines, amino acids, and thymidylate (TMP) [13]. It has been demonstrated that its absence causes the inhibition of cell growth and proliferation [14]. The synthetic pathway that allows the transformation of FA in THF is reported in Figure 1.

**Figure 2:**
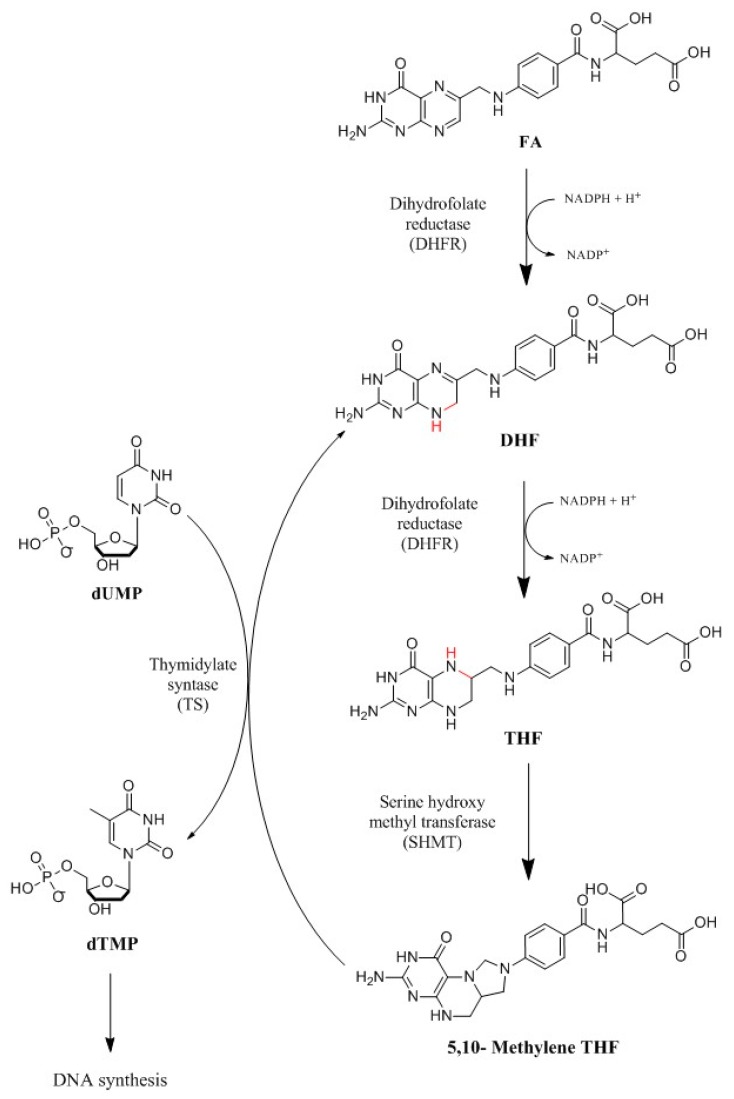
Synthetic pathway of folate metabolism. DHFR catalyzes the passage of a hydride from the cofactor nicotinamide adenine dinucleotide phosphate (NADPH), used as an electron donor, to dihydrofolate (DHF), through a protonation to produce tetrahydrofolate (THF). In particular, DHFR catalyzes the reduction of 7,8-dihydrofolate to 5,6,7,8-tetrahydrofolate using reduced NADPH as a cofactor [25]. Therefore, DHFR couples with thymidylate synthase (TS), which catalyzes the reductive methylation of deoxyuridine monophosphate (dUMP) in deoxythymidine monophosphate (dTMP) using N^5^-N^10^-methylenetetrahydrofolate (5,10-Methylene THF) as a cofactor [26].

The synthesis of folates in both eukaryotic and prokaryotic cells is strictly dependent on the activities of two enzymes: DHFR and dihydrofolate synthase (DHFS), whose inhibition leads to cell death. From a medicinal chemistry perspective, the ubiquitous enzyme DHFR is of particular interest since it is essential for folate metabolism and purine and thymidylate synthesis in cell proliferation. Poor DHFR activity causes tetrahydrofolate deficiency and cell death [15]. This mechanism is reported in Figure 2. 

**Figure 3:**
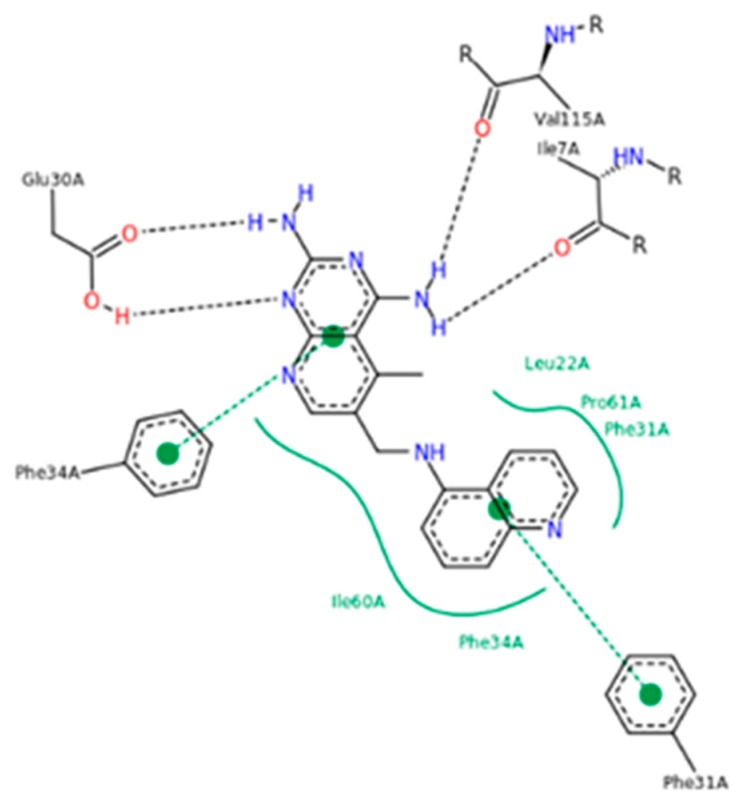
hDHFR–6-([5-quinolylamino]methyl)-2,4-diamino-5-methylpyrido[2,3-d]pyrimidine (SRI-9439) contacts (PDB ID 1KMS). Green lines show side-chain contacts, blue lines show main-chain contacts.

From a structural standpoint, DHFR is a relatively small water-soluble protein with a molecular weight of 18.000–25.000 Da. Over the years, DHFR has been extensively studied and several attempts have been made to elucidate the structure of DHFR isoforms. To date, the Protein Data Bank (PDB) has collected over one hundred structures obtained from both eukaryotic and prokaryotic organisms (humans, *Escherichia coli*, *Lactobacillus casei*, *Pneumocystis carinii*, *Micobacterium tuberculosis*, etc.), alone or in complex with different ligands [16]. Briefly, DHFR consists of eight sheets, which form a rigid skeleton: seven sheets run parallel and the other runs antiparallel. All the enzyme isoforms contain at least four α-helices intersecting in the long loops of the sheets. Furthermore, one loop forms the binding site for the substrate, while another two form the binding site of the coenzyme NADPH. It is interesting to note that DHFR has no disulfide bridges and it does not need to be coordinated by metal ions to exercise its biochemical activity [8]. An important structural element of the enzyme is the presence of the “Met20” or “loop 1” consisting of residues 9-24 [17,18,19,20,21]. It helps to stabilize the nicotinamide ring of NADPH to promote the passage of hydride from NADPH to dihydrofolate and it is able to open, close or occlude, the active site of the enzyme [22,23]. The amino acid residue Asp27 is also crucial, because it helps the protonation of the substrate and keeps it in a conformation favorable to hydride transfer [24]. The structure of the human DHFR in complex with NADPH and with the antifolate 6-([5-quinolylamino]methyl)-2,4-diamino-5-methylpyrido[2,3-*d*]pyrimidine (PDB ID: 1KMS) has been elucidated (Figure 3) [21], thus allowing the foundation for the ad hoc design of compounds specifically targeting hDHFR.

## 3. Relevance of DHFR Inhibitors in Cancer Therapy

**Figure 4:**
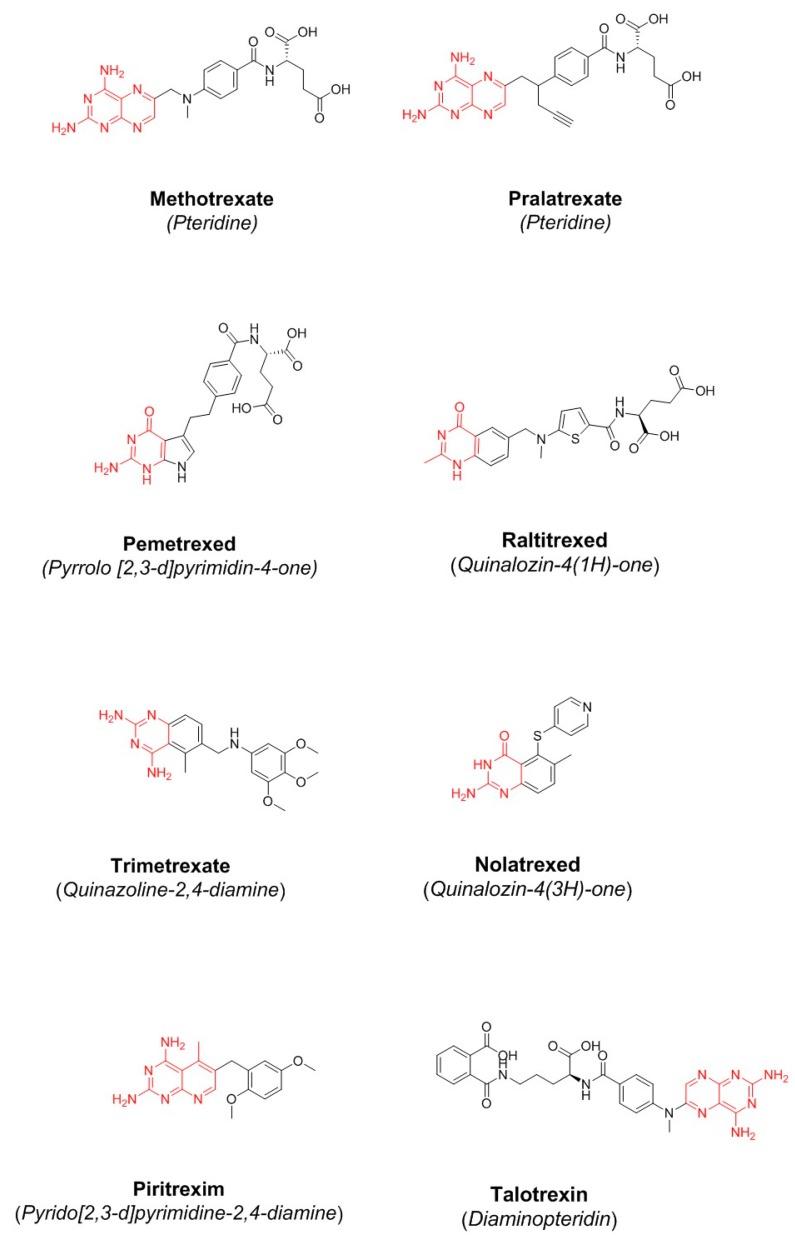
Chemical structure of some classical and non-classical antifolates.

The folate inhibitor methotrexate (Figure 4) was discovered in 1947 by Y. SubbaRow [27]. One year later, the medical doctor Sidney Farber hypothesized that cancer cells need folate to support their rapid growth and therefore that methotrexate would slow the progress of cancer. He demonstrated that methotrexate was effective in reducing symptoms in children with acute lymphoblastic leukemia [24]. A few years later, the drug entered therapy for the treatment of rheumatoid arthritis and psoriasis [28]. To date, methotrexate is widely used in humans for the treatment of some autoimmune diseases and cancer. Methotrexate is able to inhibit various isoforms of DHFR, and this low selectivity of action is responsible for the cytotoxicity towards oral mucosa and gastrointestinal (GI) tract epithelial cells, bone marrow cells, and testicular tissue involved in spermatogenesis [29,30]. 

Over the past five decades, the several attempts that have been made to obtain more effective and selective drugs have led to the discovery of the drugs reported in Figure 4 and Table 1 [31,32,33]. To date, some compounds are in clinical use: raltitrexed, pralatrexate, and pemetrexed, classified as “classical antifolates”; piritrexim, trimetrexate, talotrexin, and nolatrexed, classified as “non-classical antifolates”. From a structural point of view, classical antifolates are analogs of folate containing a pterin ring, an aromatic ring, and a glutamate tail. They do not passively diffuse across cell membranes because they possess a charged glutamate tail, so they are actively transported through the reduced folate carrier system [25,34]. Non-classical antifolates are lipophilic molecules which do not need folate transport systems and passively diffuse into cells. They are able to inhibit tumor cell growth, so they are useful for the development of new agents against cancer, as well as bacterial and parasitic infections. The last DHFR inhibitor approved by FDA was pralatrexate in 2009 [35]. It is characterized by rapid internalization into the cell, a high affinity for dihydrofolate reductase, and a good intracellular retention (Table 1).

## 4. Inhibitors of Bovine DHFR

**Figure 5:**
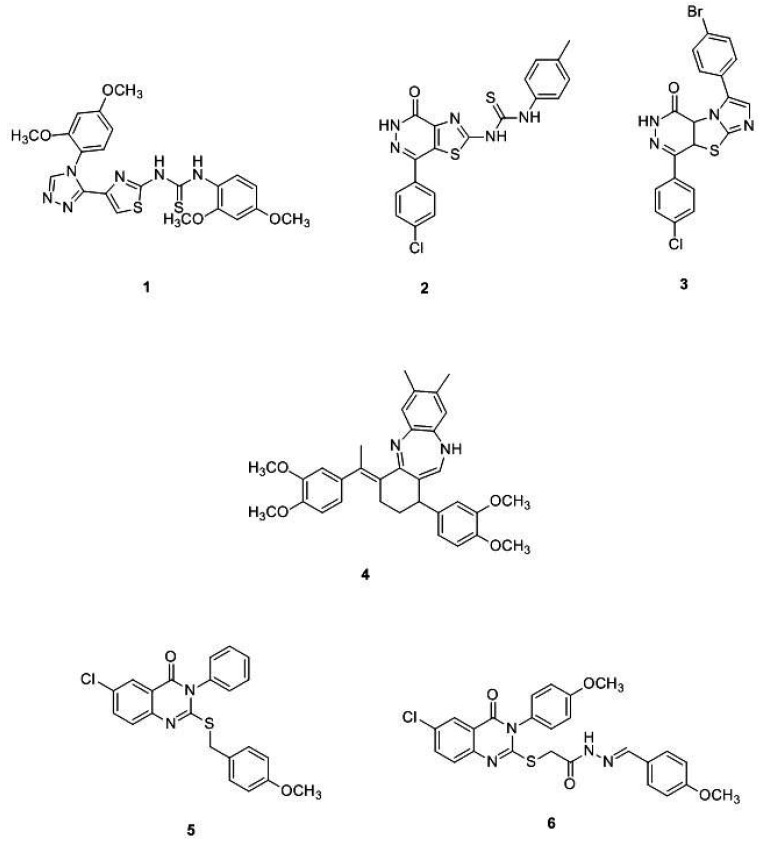
Chemical structures of some prominent bDHFR inhibitors.

To discover new effective DHFR inhibitors, a first screening using bovine DHFR (bDHFR) can be helpful in the design of drug molecules. Herein, a few examples of prominent bDHFR inhibitors are provided (Figure 5).

In 2013, Hassan et al., synthesized a new class of DHFR inhibitors based on a 5-(2-aminothiazol-4-yl)-4-phenyl-4*H*-1,2,4-triazole-3-thiol skeleton and evaluated their in vitro activity against different cancer cell lines. Among the series, compound **1** is worth mentioning since it is 2.7 times more active than the positive control methotrexate (IC_50_ = 0.08 μM). Molecular modeling docking studies with hDHFR (PDB ID: 3EIG) highlighted the amino acids Leu4 and Val1 as essential for the DHFR binding and the DHFR inhibition activity [37]. In line with these efforts, Ewida et al. studied in silico the interaction of a compound with a thiazole scaffold with an hDHFR pocket (PDB ID: 1U70). Through a detailed structural analysis, they pointed out that the thiourea moiety may act as an anchoring group, helping the thiazole scaffold to fit into the pocket with the right orientation and position. Starting from these results, the same authors designed and synthetized a new series of 2,4-substituted-1,3-thiazoles and thiazolo[4,5-*d*]pyridazine. Among all compounds tested, compound **2** (Figure 5) showed the best DHFR inhibition activity, with an IC_50_ of 0.06 μM. The high binding affinity of **2** is probably due to the additional interaction with the Phe31 and Arg22. This compound showed interesting in vitro anticancer properties, being particularly effective against the HS578T breast cancer cell line [38]. Another step of the research consisted of the preparation of a new compound series, by replacing the thiazole scaffold with imidazo[2′,1′:2,3]thiazolo[4,5-*d*]pyridazine. As a result, the potent bovine liver DHFR inhibitor **3** was discovered. This compound resulted in a promising anticancer candidate against ovarian cancer cell line OVCAR-3 and melanoma cell line MDA-MB-435, with IC_50_ values of 0.32 μM and 0.46 μM, respectively [39].

El-Subbagh et al. prepared a new series of benzodiazepines as anticancer, antiviral, and antimicrobial agents. The authors provided an efficient synthesis of a new series of tetrahydro-1*H*-dibenzo[*b,e*][1,4]diazepine analogs, presenting an α,β-unsaturated imine function. The new derivatives have been evaluated as inhibitors of bovine liver DHFR and for their antitumor activity on different cancer cell lines. Particularly, compound **4** (Figure 5) showed a good DHFR inhibition efficacy (IC_50_ = 4 nM), and was 20 times more active than the methotrexate. To rationalize the results, a molecular modelling study has been performed on hDHFR (PDB ID: 1DLS). Interestingly, the tetrahydroquinazolines derivatives positively affected the ligand-enzyme interaction, and the dibenzodiazepine ring possesses the pharmacophoric features essential for activity [40]. Recently, Shahenda et al. synthesized a new series of 2,3,6-substituted quinazolin-4(3*H*)-ones, discovering compounds **5** (Figure 5) as the most active inhibitors of DHFR (with IC_50_ =of 0.02 μM., bovine liver DHFR) [41]. Lastly, El-Gazzar et al. discovered compound **6** (Figure 5) with a 2-mercapto-quinazolin-4(3*H*)-one structure (IC_50_ =0.01 μM, bovine liver DHFR) [42].

It is of high interest that several easy-to-use kits based on ELISA or colorimetric assays, as well as mammalian (usually bovine) or human DHFR, are now commercially available, thus allowing an easy screening of potential DHFR inhibitors. No less important, DHFR enzymes belonging to different species are now well-characterized. DHFR obtained from different species (human, bovine, murine, and *E. coli* DHFR) shows a high homology degree and the variations are mainly related to the hydrophobic nature of the binding site of these enzymes [43]. 

Many recent papers are focused on the design of new compounds of different structure class with a high affinity toward human DHFR and selectivity over mammalian DHFR.

## 5. Inhibitors of Human DHFR under Preclinical Investigation

Since 2009, several DHFR inhibitors have reached clinical trials, but failed at different phases of the experimental study, owing to their high toxicity (Table 2, Figure 4). The scientific community is working hard to find new effective DHFR inhibitors, as papers and patents published so far have demonstrated. Over time, several inhibitors have been prepared, thus allowing the development of relevant SAR considerations. In this section, we will focus on compounds discovered in the period 2013–2018. Their structural features are briefly summarized hereinafter.

**Figure 6:**
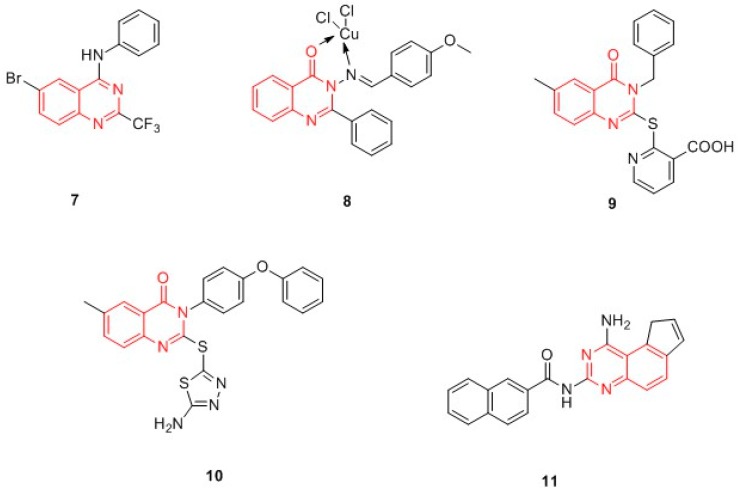
Chemical structures of quinazolines and quinazolinones hDHFR inhibitors.

Quinazolines and quinazolinones. Sahu et al. designed in silico (hDHFR, PDB ID: 1S3V) a wide series of quinazolines and quinazolinones derivatives, structurally related to raltitrexed (Figure 4). Compound **7** resulted in the most effective ligand of the quinazolinic series (Figure 6) and constituted the template for the synthesis of the quinazolinone Schiff base in the complex with different metal ions. Of note, metal-based drugs have been recognized as potential anticancer agents, since they are characterized by a low toxicity. The authors demonstrated that copper(II) derivatives are active as antagonists of hDHFR: compound **8** (Figure 6) showed an activity comparable to methotrexate towards a panel of cancer cell lines [A549 (lungs), SK-OV-3 (ovary), HCT15 (colon), K562 (leukemia), HeLa (cervix), KB (nasopharyngea), MCF7 (breast) and DU145 (prostate)] [44,45].

In 2013, Al-Omary et al. synthesized a series of 2-heteroarylthio-6-substituted-quinazolin-4-one analogues. In general, the compounds showed good IC_50_ values (ranging from 0.3 to 1.0 μM) on a panel of thirty-one cancer cell lines of leukemia; non-small cell lung cancer; CNS cancer; melanoma; and ovarian, renal, prostate, and breast cancer. Of particular interest was compound **9**, a 2-pyridinylthio-6-substituted-quinazolin-4-one derivative, which was active against different cancer cell lines [46]. Among the quinazolinones, 2-(1,3,4-thiadiazolyl- or 4-methyl-thiazolyl)thio-6-substituted-quinazolin-4-ones, prepared by Al-Rashood et al., deserve to be mentioned. Compound **10** (Figure 6) was the most active DHFR inhibitor. The SAR revealed that the substituent at positions 2, 3, and 6 in the quinazolinone nucleus contributes to the DHFR inhibition. Molecular docking studies explained the molecular bases of DHFR inhibitory activity. Docking studies (the complex hDHFR-NADPH-methotrexate was used, PDB ID: 1DLS) revealed that the amino acids Glu30, Phe31, and Phe34 are crucial for the binding interaction with the synthesized compounds. Moreover, in silico ADMET prediction studies suggested that compound **10** could be used as oral absorbing agents with a reduced toxicity [34]. In the same year, Chen et al. prepared a new series of pyrrolo[3,2-*f*]quinazoline, and all compounds were tested for their anti-breast cancer activity. Compound **11** (Figure 6) is the most interesting of the whole series, showing good activity against breast cell lines (MDA-MB-231 GI_50_ = 1.60 μM; MDA-MB-468 GI_50_ = 0.44 μM). Surprisingly, it was not able to inhibit hDHFR and therefore it has been supposed that the antiproliferative activity of compound **11** is independent of DHFR inhibition. This hypothesis was confirmed by molecular modelling studies which evidenced that the bulky naphthyl group of compound **11** is not well-accommodated in the binding pocket of hDHFR. In sum, **11** has been identified as a potent breast cancer agent independent of DHFR, and is not toxic to normal human mammary epithelial cells (HMEC) up to a 5 µM concentration [47].

**Figure 7:**
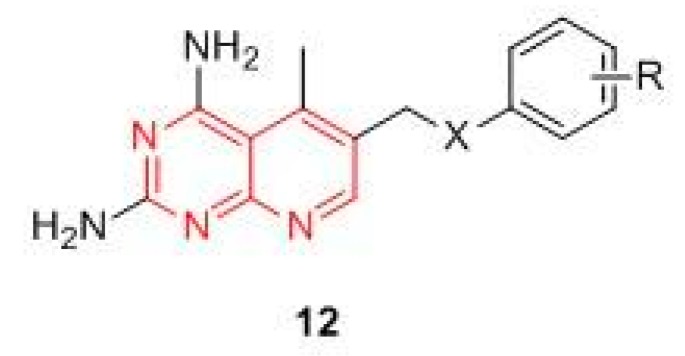
General structure of pyridopyrimidine derivatives.

Pyrimidines and pyridopyrimidines. These compounds are structurally related to trimetrexate (Figure 7), currently used for the treatment of *Pneumocycstis cariniii pneumonia* in immune compromised patients and to treat several different forms of cancer (colorectal, head, neck, and lung). Based on the assumption that the pyridopyrimidines derivatives may interact with DHFR, Du and Zhuang analyzed thirty two selected pyridopyrimidine derivatives, characterized by the general structure **12** (Figure 7), through a predictive method called the artificial neural network (ANN) to explore their potential as anticancer compounds. The study evidenced that their activity is strictly related to the nature and position of substituents on the phenyl ring, as well as the molecular charge distribution. Substituents at the phenyl moiety seem to play a crucial role in the interaction with the enzyme. Indeed, both substituents in para and ortho positions markedly decrease the binding with DHFR [48]. The results confirmed the relevance of the pyridopyrimidinic scaffold in DHFR inhibition.

**Figure 8:**
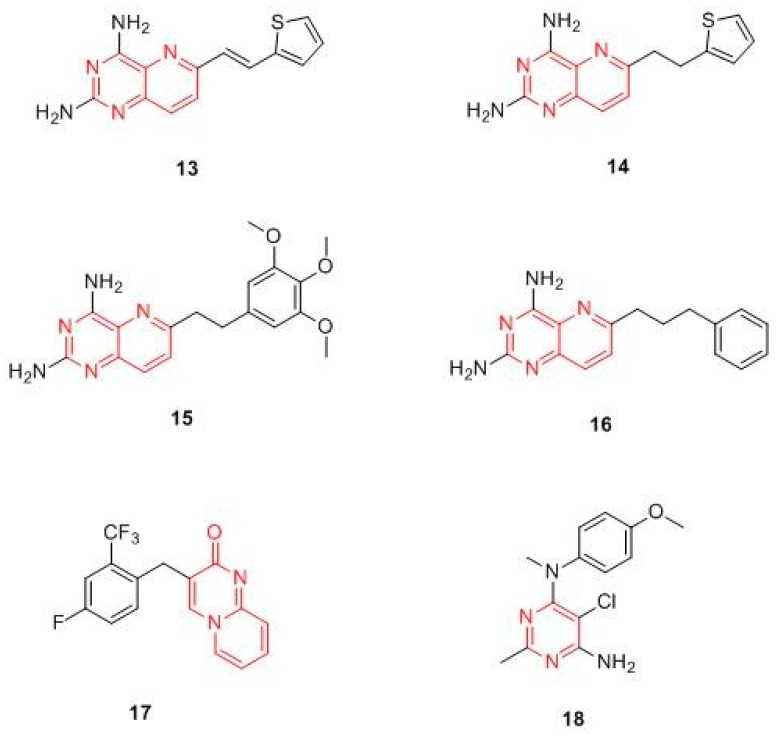
Chemical structures of pyrimidine and pyridopyrimidine hDHFR inhibitors.

Yang and Jiajia synthetized a new series of 6-substituted-2,4-diaminopyrido-[3,2-*d*]pyrimidine derivatives (Figure 8) as potential non-classical antifolate agents targeting DHFR. The compounds were evaluated for their in vitro antiproliferative activity against HL-60, HeLa, and A549 tumor cell lines and for the inhibition of recombinant human DHFR (rhDHFR). In this series, compounds **13** and **14** stand out above the others for their activity as DHFR inhibitors, with IC_50_ values of 0.59 and 0.46 μmol/L, respectively [49]. M. Wang et al. reported a new series of 2-diamino-6 substituted pyrido[3,2-*d*]-pyrimidine derivatives and evaluated their antiproliferative activity against HL-60, HeLa, A549, and H1299 human cancer cell lines. In general, the compounds exhibited micromolar antiproliferative potencies, with the exception of compound **15**, which emerged as the most potent rhDHFR inhibitor, with an IC_50_ value of 0.06 μM, and displayed interesting anticancer activity against the HL-60 cell line by inducing DNA damage due to activation of the G1/M checkpoint and the arrest in S phase [25]. In 2018, Li et al. designed and synthesized a new series of 6-substituted pyrido[3,2-*d*]pyrimidines with a three-to-five-carbon bridge. Compound **16**, belonging to the three carbon bridge series, resulted in the most potent rhDHFR inhibitor (IC_50_ = 0.06 µM), as the molecular modelling studies on human DHFR (PDB ID: 1U72) confirmed. The antiproliferative activity on three different human cancer cell lines (HL-60, HCT116, and HeLa) was then evaluated, confirming **16** as the most active compound. The authors hypothesized that its anti-proliferative activity is due to an induction of HL-60 cells apoptosis, which led to DNA damage [50].

Rapolu et al. studied the DHFR inhibitory effect and the anticancer properties of novel substituted 2*H*-pyrido[1,2-*a*]pyrimidin-2-ones. Among these, compound **17** (Figure 8) emerged as the most potent inhibitor, displaying submicromolar inhibition against DHFR (IC_50_ 3.1μM) and a good cytotoxicity (IC_50_ lower than 10 μM) against MCF-7 and SK-n-SK [51]. The series of 6-substituted pyrimidine synthesized by Gangjeeet al. should also be mentioned, since compound **18** not closely related to the other DHFR drugs appears to be a promising DHFR inhibitors candidate [52].

**Figure 9:**
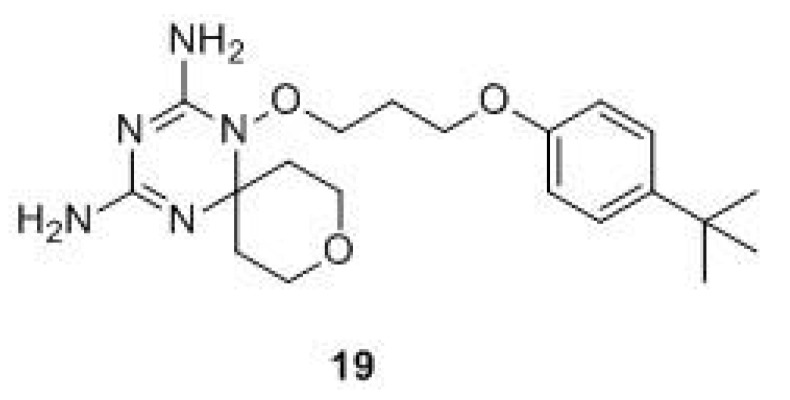
Chemical structure of triazines hDHFR inhibitors.

Triazines. As stated before, DHFR inhibitors commonly used in clinical oncology are characterized by the presence of both a nitrogen-heterocycle and an additional aromatic moiety, usually a substituted benzene ring. Starting from this observation and with the aim of introducing an element of structure novelty, Xiao-Tian Zhou et al. performed a rational drug design of novel hDHFR inhibitors and synthesized a series of compounds presenting a dihydro-1,3,5-triazine nucleus and a spirocyclic heterocycle. They analyzed the interactions of the planar bicyclic ring of hDHFR inhibitors with the residues in the hDHFR active site (PDB ID: 1U72, 1DLS, 1KMS, 1OHK, 1S3U, 2W3B, 3NTZ). The study revealed that the presence of the spiro-ring promotes the interaction with the active site of hDHFR, thanks to a shift of the flexible residue Phe31 in the DHFR pocket. The in vitro biological results demonstrated that compound **19** (Figure 9) exhibited anti-proliferative activity against tumor cell lines (HCT116, A549, HL-60, HepG2, and MDA-MB-231), with IC_50_ in the range of 3.72 nM [53].

**Figure 10:**
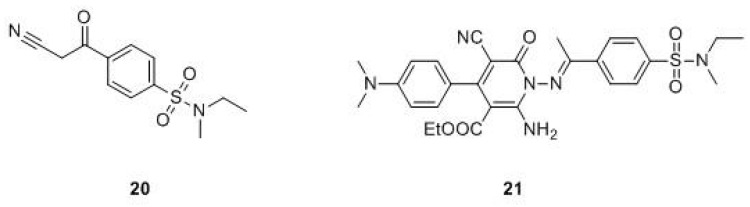
Recent hDHFR inhibitors.

Other compounds. In 2015, Al-Harbi and Bashandy synthetized a wide variety of compounds, all bearing a substituted benzenesulfonamide portion. The compounds showed high antitumor activity against the HepG2 hepatocellular carcinoma cell line (mainly compound **20**, Figure 10), with interesting IC_50_ (1.38-39.70 μg/mL) and selectivity index (SI) (2.06–70.92) values when compared with methotrexate as a positive control (IC_50_ = 3.21 μg/mL, SI = 13.30). Molecular docking studies evidenced that some compounds exhibit strong interactions with the DHFR pocket (PDB ID: 4DFR) [54].

In 2017, Debbabi et al. described some new pyridin-N-ethyl-N-methylbenzenesulfonamides as efficient anticancer agents against the MCF-7 breast cancer cell line, showing IC_50_ values in the range of 7.68-22.7 µM with a selectivity index in the range of 4.71-9.72. Docking studies confirmed the interaction of **21**, the best compound of the series in terms of activity, with DHFR active sites (PDB ID: 4DFR) [55].

**Figure 11:**
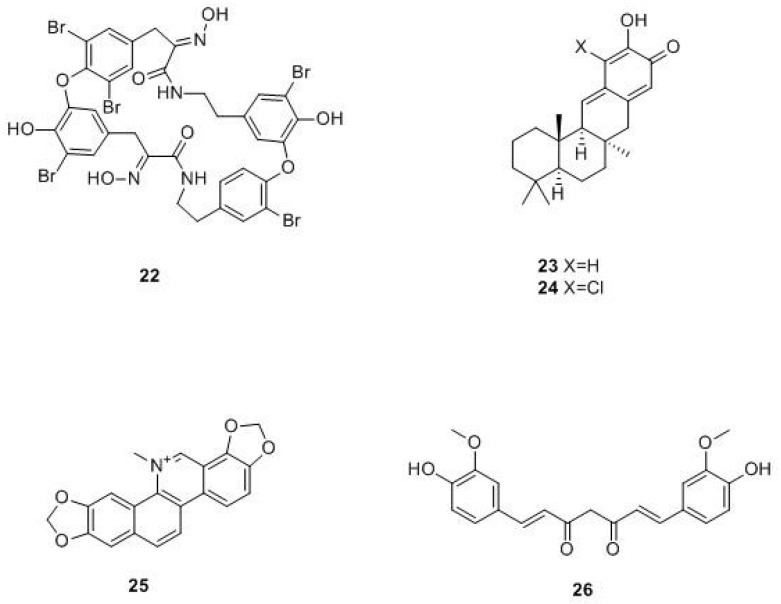
Natural compounds active against the DHFR enzyme.

Natural compounds. One of the most relevant limiting factors in the drug-discovery process is organic synthesis. Despite the advances in this research field, the number of compounds that medicinal chemists can synthesize is still limited and experimental synthesis is still considered the bottleneck in the discovery of novel bioactive compounds [56]. From this perspective, nature is a limitless source of compounds with unique chemical skeletons and potent bioactivities. Nevertheless, only a few human DHFR inhibitors have been isolated from nature, and in particular, from marine and vegetal sources. Regarding marine sources, interesting compounds have been extracted from sponges. Among these, bastadin (**22**, Figure 11), a metabolite of *Ircinia muscarum* able to inhibit hDHFR with an IC_50_ value of 2.5 µg/mL, and Puupehenone (**23**) and 21-chloro Puupehenone (**24)** isolated from a *Verongid* sponge, have shown good DHFR inhibitory activity (IC_50_ = 5 µg/mL) [57].

Among the novel DHFR inhibitors from the plant kingdom, recently, Kalogris et al. identified Sanguinarine (**25**), a natural benzophenanthridine alkaloid derived from the root of *Sanguinaria canadensis*, as a potent inhibitor of DHFR, able to induce a modest and significant impairment of DHFR enzymatic activity [58]. Sanguinarine showed a remarkable anti-neoplastic activity by inducing apoptosis in various human cancer cell lines, including human breast cancer ones. The authors demonstrated that the anticancer activity of sanguinarine is DHFR-mediated. The inhibitory effect on this crucial target has been demonstrated in A17 cells and in MTX-resistant MDA-MB-231 cells, where the alkaloid reduced the enzyme activity to 30% and 50%, respectively. Notwithstanding, sanguinarine seems to be capable of reducing the enzyme activity also in the BLBC cells, resistant to MTX.

Within a wider research project aimed at discovering the antitumor and antifolate activity of a series of popular medicinal plants, Albalawi et al. identified *Caralluma sinaica* and *Fagonia tenuifolia* as potential anticancer-drug sources worthy of further investigation. The best results in terms of in vitro inhibition of DHFR activity were shown by *Sonchus oleraceus* extracts (0.06 μg/L) and *Caralluma sinaica* extracts (0.10 μg/L). Since *Caralluma sinaica* extracts are active in both antitumor and antifolate assays, they could assume that the anticancer activity of this plant proceeds through DHFR inhibition. Thus, this study provides scientific support for the use of *Caralluma sinaica* as a medicinal anticancer plant [59].

Lastly, also curcumin, which therapeutic potential against several diseases, including cancer, has been identified as being able to prominently bind molecules of the DHFR enzyme by Yahya et al. In detail, they predicted the interaction of curcumin by molecular docking on the DHFR active site (PDB ID: 1DRE). Surprisingly, the analysis revealed that curcumin binds to DHFR with a strong affinity, even comparable to that of methotrexate, as confirmed by the values of free binding energy (ΔG = −9.02 kcal/mol; Ki = 243 nM for curcumin and ΔG = −8.78 kcal/mol; Ki = 363 nM for methotrexate). These results suggest that curcumin may interact with the enzyme with similar modes of action of known DHFR inhibitors. Furthermore, curcumin (**26**) is able to optimize the interactions on both sides of the enzyme pocket because it binds to a folded conformation in the active site of DHFR due to its structural flexibility. It also establishes van der Waals interactions with some active enzyme residues and a π-π interaction with the phenyl ring of Phe34 and the phenyl ring of curcumin, in the active site pocket. Further studies are necessary for confirming this mechanism of action [60].

As highlighted in the previous paragraphs, different research groups are involved in the design and synthesis of novel DHFR inhibitors as effective drugs for counteracting cancer. In vitro biological properties and corresponding SAR of investigated compounds clearly correlate the DHFR inhibition with the anticancer effect. Several scaffolds have been identified, opening the door for the development of better drugs. As previously outlined, most SAR studies suggest that at least the presence of a heterocyclic cycle is necessary for an anticancer effect, with the only exception of sulfonamide derivatives.

## 6. New Strategies in DHFR Drug Discovery

**Figure 12:**
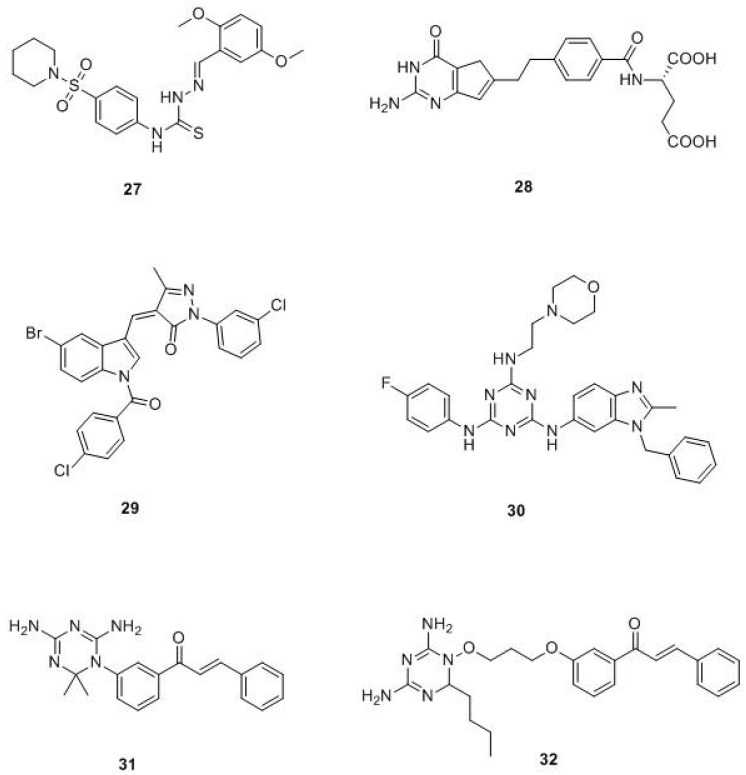
Chemical structures of dual inhibitors.

It is well-known that the use of drugs in combination, compared to the mono-therapy approach, may have synergistic or additive efficacy against cancer. Accordingly, hybrid compounds are a good strategy for cancer treatment. Arooj et al. discovered potential dual inhibitors of human thymidylate synthase (hTS) and hDHFR, by developing a pharmacophoric model with the chemical features necessary for the dual inhibition of target enzymes (hDHFR, PDB ID: 1U72; hTS, PDB ID: 1HVY). Afterwards, they applied a virtual screening approach, starting from a drug-like database. Once compounds with different scaffolds potentially able to interact with both targets have been selected, optimization studies have been performed, leading to dual compounds with excellent binding characteristics towards the target proteins (compound **27**, Figure 12) [61].

In 2017, Tian et al prepared a new series of 6-substituted pyrrolo[3,2-*d*]pyrimidines acting as dual inhibitors on TS and DHFR. Molecular modelling studies demonstrated that all synthetized compounds interact on the active sites of hTS (PDB ID: 1JU6) and hDHFR (PDB ID 1U72) enzymes with high binding affinity values. The enzyme assay on DHFR established that the most active compound was **28** (Figure 12), with an inhibitory ratio of 66.7% at 100 µM. Among all synthesized compounds, only **28** showed a good anti-proliferative activity against A549, NCI-H1299, and HL60 tumor cell lines, with GI_50_ values of 0.73, 1.72, and 8.92 μM, respectively. Moreover, **28** has determined G2/M phase arrest on cell cycle distribution in A549 cells [62].

Shaveta and Singh synthesized a series of indole, pyrazole, and barbituric acid conjugates as potential anticancer agents. Of particular interest is compound **29** (Figure 12), which is active against non-small cell lung cancer NCI-H522. This compound interacts with enzymes involved in the cancer diffusion process like DHFR, RNR (ribonucleotide reductase), TS, and TP (thymidylate phosphorylase). Molecular docking studies on the hDHFR pocket (PDB ID: 3GHW) revealed that compound **29** established H-bonds between pyrazolic nitrogen and the Gln35 residue of the α-helix of the enzyme, so it was placed in the active site of the enzyme. Moreover, the indole moiety established a favorable hydrophobic interaction, while the p-chlorobenzoyl group established a polar interaction. These results showed that the indole moiety aligns parallel with the β sheet, while the p-chlorobenzoyl fragment overlaps with the other β-sheet, revealing the compatibility of compound **29** towards the active site of DHFR [63].

In 2016, Singla et al. synthesized a new series of triazine-benzimidazole analogues with a 4-fluoroaniline moiety. The in vitro effect of all compounds on human cancer cell lines has been evaluated and DNA intercalation studies performed. All compounds exhibited intercalation properties with ct-DNA (calf thymus-DNA) and they also exerted significant DHFR inhibitory activity, with IC_50_ values ranging from 0.11 to 42.4 μM. SAR studies revealed that the nature of the substituent in the C_2_- and C_6_- positions of triazine nucleus influences the activity, and among all, compound **30** turned out to be the most active hDHFR inhibitor, with an IC_50_ value of 2.0 nM [64].

In the same year, Ng et al. evaluated the effect of the co-administration of dihydrotriazine acting as DHFR inhibitors and chalcones acting as thioredoxin reductases (TrxR) inhibitors for the treatment of breast (MCF-7) and colorectal (HCT116) carcinoma cells. The combination therapy showed a synergic anti-proliferative effect. Starting from these results, the authors synthesized four dihydrotriazine-chalcone hybrid compounds. Among these, compound **31** [DHFR IC_50_ = 2.4 µM, TrxR IC_50_ (60 min) = 10.1 µM] demonstrated in vitro inhibition of both DHFR and TrxR (Figure 12) [65]. The same authors synthesized fifteen new compounds, connecting the two scaffolds through a diether linker (Figure 12), successfully obtaining dual inhibitors against DHFR and TrxR, as demonstrated by in vitro enzyme assays with IC_50_ values ranging from 2.6–53.4 nM on DHFR and IC_50_ values ranging from 3.8–32.4 μM on TrxR. All compounds showed cytotoxic activity against HCT116 and MCF-7 cancer cell lines (methotrexate sensitive), exhibiting a biphasic dose-response curve. GI_50_ range values were from 0.011 to 2.18 μM and LC_50_ range values were from 9.7 to 81.9 μM. It is worth noting that the length of the diether linker influenced the growth inhibitory effects: compound **32** with a linker length of three carbon atoms showed the strongest growth inhibitory activities (GI_50_ of 0.026 μM on HCT116 cells andGI_50_ of 0.008 μM on MCF-7 cells) [66]. 

Lastly, Hsieh et al. proposed a novel mechanism to induce DHFR degradation through cofactor depletion in neoplastic cells by the inhibition of NAD kinase. The authors identified an inhibitor of NAD kinase, thionicotinamide adenine dinucleotide phosphate (NADPS), which accelerated the degradation of DHFR and inhibited cancer cell growth. They demonstrated that the combination treatment of NADPS with methotrexate displayed a synergic effect in metastatic colon cancer cell lines and proposed NAD kinase as a valid target for further inhibitor development for cancer treatment [67].

## 7. Conclusions

The role of the DHFR enzyme as a therapeutic target in cancer treatment has been well-recognized for several years and the discovery of new compounds able to inhibit this enzyme continues to attract the interest of medicinal chemists. The analysis of the scientific literature of the last six years showed that all novel DHFR inhibitors (both synthetically or naturally derived) are characterized by the presence of heterocyclic moieties in the structure, with the only exception of sulphonamides. Lastly, dual compounds, combining DHFR with other folate receptors (FRs) inhibition and endowed with interesting antitumor properties, have recently been discovered. In this scenario, multi-target drugs are a promising approach for discovering anti-cancer agents. A new generation of DHFR inhibitors able to simultaneously bind different targets may be designed, which could lead to novel effective drugs.

## Figures and Tables

**Table 1:** DHFR inhibitors that have been approved [36].

Antifolate	Status *	Indication	Toxicity
Methotrexate	Approved by FDA and EMA in 1985	Treatment of lymphoma, acute lymphoblastic leukemia and osteosarcoma	Symptoms of overdose include bone marrow suppression and gastrointestinal side effects
Raltitrexed	Approved by EMA in 1998	Treatment of malignant colorectal cancer, but its utilization in mainly limited to patients who are intolerant to 5-fluorouracil	Gastrointestinal and hematologic side effects
Pemetrexed	Approved by FDA and EMA in 2001	First-line treatment for advanced non-squamous-cell lung cancer and pleural mesothelioma in combination with cisplatin	Neutropenia, leukopenia, anemia, stomatitis and infection
Pralatrexate	Approved by FDA and EMA in 2009	Treatment of relapsed or refractory peripheral T-cell lymphoma (TCL)	Mucositis

* Source: DrugBank https://www.drugbank.ca/drugs/DB03695.

**Table 2:** DHFR inhibitors withdrawn from clinical trials.

Antifolate	ClinicalTrials.gov RECORD ID *	Status	Toxicity
Nolatrexed	NCT00012324	Phase 3 study in unresectable hepatocellular carcinoma (HCC) has been completed (2005)	Nausea, vomiting, stomatitis, erythematous maculopapular rash, thrombocytopenia and neutropenia
Piritrexim	NCT00002914	Phase 2 study in advanced cancer of the urinary tract has been completed (2004)	Leukopenia, thrombocytopenia, mucositis
Talotrexin	NCT00088023 NCT00112060 NCT00129558 NCT00458744	It has been suspended in phase 1 in the treatment of solid tumors (2005). It was withdrawn in phase 1 in the treatment of brain and central nervous system tumors, and malignant lymphomas (2008). It was withdrawn in phase 2 in the treatment of non-small-cell lung carcinoma (NSCLC) and leukemia (2011)	Neurotoxic effect such as fatigue and hypoxia. At higher and cumulative doses, it may produce fatal leukoencephalopathy

* Source: https://clinicaltrials.gov/.

## Abbreviations

FA	Folic acid
THF	tetrahydrofolate
DHFR	dihydrofolate reductase enzyme
hDHFR	human DHFR
bDHFR	bovine DHFR
rhDHFR	recombinant human DHFR
hTS	human thymidylate synthase
NADPH	nicotinamide adenine dinucleotide phosphate
dUMP	dihydrofolate synthase (DHFS), deoxyuridine monophosphate
dTMP	deoxythymidine monophosphate
5,10-Methylene THF	N^5^-N^10^-methylenetetrahydrofolate
TrxR	thioredoxin reductases
FRs	folate receptors
ct-DNA	calf thymus-DNA
RNR	ribonucleotide reductase
TS	thymidylate synthase
TP	thymidylated phosphorylase
MTX	methotrexate
ANN	artificial neural network
PDB	Protein Data Bank
Asp127	Aspartate 127
Glu30	Glutamate 30
Phe31	Phenylalanine
ELISA assay	enzyme-linked immunosorbent assay
FDA	U.S. Food and Drug Administration
EMA	European Medicines Agency
ADMET	absorption, distribution, metabolism, excretion, toxicity
OVCAR-3	ovarian cancer cell line
MDA-MB-435	melanoma cell line
NSCLC	non-small-cell lung carcinoma
HCC	hepatocellular carcinoma
A549	adenocarcinomic human alveolar basal epithelial cells
SK-OV-3	human ovary cancer cell line
HCT15; HCT116	human colon adenocarcinoma colorectal adenocarcinoma
K562	human chronic myeloid leukemia cell
HeLa	human of immortal cervical cancer cell
KB	keratin-forming tumor cell line HeLa
MCF-7; MDA-MB-231; MDA-MB-468	human breast cancer cell line
DU145	human prostate cancer cell line
HMEC	normal human mammary epithelial cells
HL-60	human caucasian promyelocytic leukemia cell line
NCI-H1299; NCI-H522	human non-small cell lung carcinoma cell line
HepG2	human liver cancer cell line
SK-n-SH	human neuroblastoma
A17	amacrine cells in mammalian retina
SAR	structure activity relationships
